# Hypoglycemia Occurrence and Risk Factors in Insulin Independent Patients After Total Pancreatectomy With Islet Autotransplantation

**DOI:** 10.1111/ctr.70549

**Published:** 2026-04-29

**Authors:** Azza Hassaan, Anne Eaton, Niveditha Jagadesh, Guru Trikudanathan, Martin Freeman, Elissa Downs, Srinath Chinnakotla, David Martin, Karthik Ramanathan, Gregory J. Beilman, Melena D. Bellin

**Affiliations:** ^1^ Department of Pediatrics University of Minnesota Medical School Minneapolis Minnesota USA; ^2^ Division of Biostatistics and Health Data Sciences University of Minnesota School of Public Health Minneapolis Minnesota USA; ^3^ Department of Surgery University of Minnesota Medical School Minneapolis Minnesota USA; ^4^ Department of Internal Medicine University of Minnesota Medical School Minneapolis Minnesota USA

**Keywords:** acute pancreatitis, autoislet, autotransplant, chronic pancreatitis, diabetes, IAT, pancreatitis, TPIAT, type 3c diabetes

## Abstract

**Background:**

Total pancreatectomy with islet auto‐transplantation (TPIAT) alleviates pain and preserves islet function in patients with severe pancreatitis. Although approximately one third of recipients achieve insulin independence, spontaneous hypoglycemia has been observed in this population. Its frequency, risk factors, and management remain poorly characterized.

**Methods:**

Using prospectively collected data on insulin‐independent (TPIAT) recipients at the University of Minnesota (2010–2023), we compared patients who developed hypoglycemia (defined as documented blood glucose < 70 mg/dL) to those with no reported hypoglycemia. Demographic, clinical, surgical, and metabolic variables were evaluated, and treatment strategies were reviewed.

**Results:**

Among 503 TPIAT recipients, 156 (31%) achieved insulin independence; of these, 45 (28.8%) experienced hypoglycemia, including 32 (20.5%) with level 2 hypoglycemia (< 54 mg/dL). Severe hypoglycemia events requiring external assistance occurred in 25% of affected individuals. Hypoglycemia developed a mean of 2.4 ± 2.2 years posttransplant, most commonly postprandial or exercise‐related. Patients who developed hypoglycemia were older at the time of TPIAT (*p* = 0.036) and had higher preoperative BMI (*p* = 0.016) and a greater prevalence of malabsorptive disorders (*p* < 0.001), whereas islet yield and metabolic testing results were similar between patients with and without hypoglycemia. Most patients with hypoglycemia (78%) were managed with non‐pharmacological interventions (dietary, continuous glucose monitoring); pharmacologic therapies included acarbose (22%), diazoxide (13%), and minidose glucagon (7%).

**Conclusions:**

Spontaneous hypoglycemia is a frequent, clinically relevant complication among insulin‐independent TPIAT recipients. Older age, higher BMI, and malabsorptive disorders were associated with increased susceptibility. Routine surveillance, dietary counseling, and continuous glucose monitoring (CGM) are common components of management.

**Abbreviations**
AUC: area under the curve
BMI: body mass index
CFTR: cystic fibrosis transmembrane conductance regulator
CGM: continuous glucose monitor
EMR: electronic medical record
HbA1c: hemoglobin A1c level
HOMA‐IR: homeostatic model assessment of insulin resistance
IEQ/kg: islet equivalents per kilogram body weight
MMTT: mixed meal tolerance test
prss1: cationic trypsinogen gene
SPINK1: serine peptidase inhibitor kazal‐type 1 gene
TPIAT: total pancreatectomy with islet autotransplant

## 1 Introduction

Total pancreatectomy with islet auto‐transplantation (TPIAT) is a surgical procedure designed to treat patients suffering from debilitating chronic pancreatitis or recurrent acute pancreatitis when other medical and endoscopic treatments fail to provide adequate relief.[1] This procedure involves the complete removal of the pancreas to alleviate intractable abdominal pain, followed by isolation and transplantation of the patient's own islet cells to preserve endocrine function and reduce or prevent surgically induced diabetes. While approximately 30% of patients achieve insulin independence following TPIAT, [2, 3] limited published data suggest that a subset of these insulin‐independent individuals experience episodes of hypoglycemia, sometimes severe, despite not requiring exogenous insulin [4].

The mechanisms underlying post‐TPIAT hypoglycemia in insulin‐independent patients remain unclear. Proposed contributors include Roux‐en‐Y–related alterations in vagal innervation, nutrient absorption, and hormonal regulation, as well as changes in insulin sensitivity and incretin effects [4, 5]. In addition, hypoglycemic clamp studies in insulin independent TPIAT patients suggest an impaired glucagon response to hypoglycemia. [6]

While hypoglycemia in this population has been reported, prior publications are limited to small cohorts and case series, and lack systematic characterization of hypoglycemia. [4, 7, 8, 9] Post TPIAT hypoglycemia can adversely impact patient quality of life and is often complex and challenging to manage clinically. Pharmacologic and non‐pharmacologic treatment strategies have been limited to a few case reports, including dietary intervention, and medications including acarbose, diazoxide, and minidose glucagon.[8, 10, 11] Furthermore, there is limited research to determine risk factors which may identify patients that require closer monitoring.

We utilized our large TPIAT patient population to assess the frequency of post‐TPIAT hypoglycemia reported among a large cohort of insulin‐independent patients, the treatment modalities used in clinical practice, and potential risk factors that may predict those at higher risk for developing hypoglycemia.

## 2 Methodology

### 2.1 Study Design

We analyzed data from a single‐center prospective cohort study of individuals who underwent TPIAT at the University of Minnesota between 2010 and 2023. All patients included in this analysis are enrolled in a long‐term cohort study for TPIAT outcomes at our center. The study was reviewed and approved by the University of Minnesota IRB, and informed consent (or parental consent/ patient assent) was obtained for each participant. Individuals with a partial pancreatectomy with IAT were excluded.

### 2.2 Study Population

Eligible patients for this analysis were individuals who achieved insulin independence (consistently off for at least a three‐month period) following TPIAT (*N* = 156). Patients were classified as having recurrent hypoglycemia in the outpatient setting, or no reports of hypoglycemia. Documented hypoglycemia was defined as a blood glucose level < 70 mg/dL, confirmed via CGM, glucometer records, or electronic medical records. Patients whose hypoglycemic events were captured by CGM were not routinely verified with glucometer results in the medical records, as patient clinical evaluation in the setting of hypoglycemia varied between providers. Level 2 hypoglycemia was defined as a blood glucose level < 54 mg/dL. Severe hypoglycemia was defined as any episode associated with loss of consciousness, seizure, emergency glucagon administration, or paramedic intervention. Hypoglycemia history was captured using two data sources: (1) Clinical encounters for management of hypoglycemia in the electronic medical record including outpatient clinic visits; and/or (2) Participant reported hypoglycemia and severe hypoglycemia that was collected from yearly study questionnaires. Patients were 1–15 years out from TPIAT at the time of database lock, and all available records were used to classify patients as insulin independent, or not, and as experiencing hypoglycemia, or not.

### 2.3 Medical History and Surgical Details

Demographic variables, clinical history, and surgical characteristics were collected from our study database and electronic medical records. Preoperative body mass index and gastrointestinal comorbidities were collected. Surgical variables included islet mass transplanted per kilogram body weight, anatomical features (e.g., pylorus preservation), site of islet infusion, and presence of microbial contamination in islet preparations. For those diagnosed with hypoglycemia, potential contributors to hypoglycemia, including adrenal insufficiency diagnoses and use of non‐insulin diabetes medications, were also evaluated.

### 2.4 Laboratory Data and Glycemic Monitoring

Patients at our center have routine preoperative and yearly testing for islet function with a MMTT. In our population, MMTT was performed by ingestion of a Boost HP beverage with glucose and C‐peptide measured before Boost HP and at 1 h and 2 h after. Hemoglobin A1c was collected with annual labs. HOMA‐IR, was calculated as 1.5 + (fasting plasma glucose × fasting C‐peptide) / 2800 for a surrogate measure of insulin sensitivity. For the purpose of the current analyses, we collected these data at the nearest time after hypoglycemia onset was reported for the patients with hypoglycemia and at the approximately 2 ‐year follow up visit for patients without hypoglycemia. The 2‐year labs were selected based on: (1) Our observation that this was close to the same average time at which hypoglycemia is reported; (2) Relatively complete data are available at this time post‐TPIAT.

### 2.5 Treatment of Hypoglycemia

Management strategies were reviewed from the EMR. Treatment modalities for hypoglycemia were classified into dietary/lifestyle interventions and pharmacological strategies, including microdosed glucagon (administration of small, frequent doses of glucagon to compensate for deficient endogenous glucagon secretion), diazoxide (which inhibits insulin secretion by opening ATP‐sensitive potassium channels in pancreatic beta cells), and acarbose (an alpha‐glucosidase inhibitor that slows carbohydrate absorption in the gut, reducing postprandial glucose spikes). CGM use was categorized based on its application for routine monitoring versus diagnostic evaluation. Where feasible, we also collected treatment responses based on patient and provider perception and CGM data.

### 2.6 Statistical Analyses

Participant characteristics were summarized by group (any hypoglycemia, level 2 hypoglycemia, no reported hypoglycemia) using the mean and standard deviation for numeric variables and the frequency and percentage for categorical variables. Groups were compared using t‐tests for numeric variables and Chi‐squared tests and Fisher's exact tests for categorical variables. *P*‐values less than 0.05 were considered significant. All statistical analysis was performed in R (R Foundation for Statistical Computing, Vienna, Austria).

## 3 Results

### 3.1 Occurrence of Hypoglycemia in Insulin Independent Patients after TPIAT

Of 503 patients who underwent TPIAT and were reviewed for the current study, 156 (31%) achieved insulin independence. Among these, 45 individuals (28.8%) experienced documented hypoglycemia (blood glucose < 70 mg/dL) while off insulin therapy. Of these, 32 patients (71.1% of the hypoglycemia cohort or 20.5% of all insulin independent patients) met criteria for level 2 hypoglycemia, with blood glucose levels < 54 mg/dL. Insulin‐independent hypoglycemia was first reported at a mean of 2.38 ± 2.16 years after TPIAT with mean age of 34 ± 16 years (range 6–69). One patient in the hypoglycemia group was receiving non‐insulin antihyperglycemic medication, with a sodium‐glucose cotransporter two inhibitor. Four patients with hypoglycemia received clinical diagnoses of possible adrenal insufficiency and showed improvement following treatment.; however, this diagnosis was considered questionable by endocrine providers in two of the four cases — in these two cases, the morning adrenocorticotropic hormone or cortisol were low, but cosyntropin stimulation testing was normal. Two individuals in the hypoglycemia cohort had a history of evaluation for unexplained hypoglycemia prior to surgery.

### 3.2 Clinical Significance and Severity of Hypoglycemia post TPIAT Surgery

Of the 45 cases with hypoglycemia, 11 (25% of the hypoglycemia cohort, or 7% of all insulin independent patients) reported severe hypoglycemic events, defined as episodes associated with loss of consciousness, seizure activity, or the need for emergency intervention, with most experiencing one or two episodes. The timing of hypoglycemic episodes was reported as follows (with some patients reporting multiple types of hypoglycemia): postprandial in 24 cases (53.3%), exercise‐related in 26 cases (57.8%), nocturnal in five cases (11.1%), fasting‐related in four cases (8.9%), and spontaneous (not clearly associated with identifiable triggers) in three cases (6.7%). Among the 36 patients who had available data about hypoglycemia awareness recorded in clinical encounters, normal hypoglycemia awareness was reported in 67% of patients, while 33% reported reduced or absent awareness of symptoms.

### 3.3 Pre‐TPIAT Clinical, Laboratory, and Surgical Parameters Associated with Post TPIAT Hypoglycemia

We compared the pre‐TPIAT and surgical variables between all patients with hypoglycemia and those with no reported hypoglycemia Table 1. The hypoglycemia cohort was older at the time of surgery (31 ± 16 years vs. 25 ± 16 years for controls, *p* = 0.036). Hypoglycemia cases had a higher BMI (23.7 ± 4.3 kg/m^2^ vs. 21.6 ± 5.8 kg/m^2^, *p* = 0.016), and etiology of pancreatitis differed between the two groups (*p* = 0.015), particularly notable for more idiopathic or obstructive disease in the hypoglycemia cohort, and more genetic disease in controls. *SPINK1* mutations were less frequent in the hypoglycemia group (*p* = 0.012).

**TABLE 1:** Pre‐TPIAT and surgical variables in insulin independent patients with hypoglycemia (<70 mg/dl) or level 2 hypoglycemia (<54 mg/dl) versus no reported hypoglycemia post‐tpiat, data are displayed as mean (SD) or as *n* (%).

Characteristic	No reported hypoglycemia *N* = 111	Any hypoglycemia (Level 1 or Level 2) *N* = 45	*p*‐value^*^	Level 2 hypoglycemia *N* = 32	*p*‐value^**^
**Age at TPIAT (years)**	25 (16)	31 (16)	**0.036**	32 (13)	**0.025**
**Age group at TPIAT**			0.062		**0.007**
Adult (≥18 years)	61 (55%)	32 (71%)		26 (81%)	
Child (<18 years)	50 (45%)	13 (29%)		6 (19%)	
**Sex, female**	82 (74%)	34 (76%)	0.8	26 (81%)	0.4
**Preoperative BMI (kg/m^2^)**	21.6 (5.8)	23.7 (4.3)	**0.016**	24.7 (4.0)	**<0.001**
**Etiology**			**0.015**		**0.019**
Idiopathic	19 (17%)	16 (36%)		12 (38%)	
Familial/genetic	61 (55%)	14 (31%)		9 (28%)	
SOD/biliary	12 (11%)	9 (20%)		7 (22%)	
Annular pancreas/ Pancreas divisum	16 (14%)	4 (8.9%)		3 (9.4%)	
Other	3 (2.7%)	2 (4.4%)		1 (3.1%)	
**Genetic mutations**					
CFTR	30 (27%)	8 (18%)	0.2	6 (19%)	0.3
PRSS1	27 (24%)	6 (13%)	0.13	2 (6.3%)	**0.025**
SPINK1	19 (17%)	1 (2.2%)	**0.012**	1 (3.1%)	**0.046**
**Malabsorption/maldigestion comorbidity**	11 (9.9%)	15 (33%)	**<0.001**	11 (34%)	**0.002**
**Gastroparesis**	39 (35%)	21 (47%)	0.2	18 (56%)	**0.032**
**Pre‐TPIAT chronic opioid use**	58 (52%)	30 (67%)	0.10	25 (78%)	**0.005**
**HbA1c level (%)**	5.17 (0.32)	5.15 (0.41)	0.8	5.14 (0.42)	0.8
**MMTT glucose and C‐peptide**					
Fasting glucose (mg/dL)	87 (8)	89 (8)	0.2	89 (9)	0.4
1 h glucose (mg/dL)	86 (24)	84 (17)	0.6	84 (19)	0.7
2 h glucose (mg/dL)	88 (14)	86 (18)	0.4	85 (22)	0.4
2 h AUC glucose (mg/dL^*^h)	173 (28)	172 (27)	0.9	172 (31)	0.9
Any glucose <70 mg/dL during testing	24 (24%)	12 (30%)	0.5	9 (33%)	0.3
Fasting C‐peptide (ng/dL)	1.74 (1.34)	2.00 (1.27)	0.3	2.08 (1.37)	0.2
1 hC‐peptide (ng/dL)	5.18 (3.88)	6.72 (6.23)	0.2	6.87 (7.23)	0.3
2 h C‐peptide (ng/dL)	3.60 (2.22)	4.63 (3.10)	0.059	4.97 (3.45)	0.052
2 h AUC C‐peptide (ng/dL^*^h)	7.8 (5.0)	10.0 (8.0)	0.13	10.3 (9.2)	0.2
**HOMA‐IR**	1.55 (0.05)	1.56 (0.05)	0.3	1.56 (0.05)	0.3
**Surgery and islet details**					
Pylorus sparing	106 (95%)	39 (87%)	0.079	28 (88%)	0.11
Transplanted islet mass (IEQ/kg)	5894 (2,495)	5820 (2,089)	0.9	5917 (2,170)	>0.9
Extra‐portal (second site) for islet transplant			0.5		0.4
None, all islets went intraportal	84 (76%)	30 (67%)		22 (69%)	
Intraperitoneal	24 (22%)	13 (29%)		8 (25%)	
Omentum	2 (1.8%)	2 (4.4%)		2 (6.3%)	
Small bowel mesentery	1 (0.9%)	0 (0%)		0 (0%)	
Microbial culture positive, pancreas preservation solution	62 (56%)	19 (42%)	0.12	18 (56%)	0.2
Microbial culture positive, islet product	35 (32%)	7 (16%)	**0.042**	4 (13%)	**0.033**

*Note:* Missing laboratory data: HbA1c missing in three patients without hypoglycemia; Glucose is missing fasting for one participant with hypoglycemia and four patients without hypoglycemia; 1 h seven patients with hypoglycemia and eight patients without hypoglycemia; 2 h three patients with hypoglycemia and 10 patients without hypoglycemia; C‐peptide is missing fasting for three patients with hypoglycemia and five patients without hypoglycemia; 1 h for nine patients with hypoglycemia and eight patients without hypoglycemia; and 2 h for four patients with hypoglycemia and 11 patients without hypoglycemia. AUC calculations could not be completed due to missing data in seven patients with hypoglycemia /12 patients without hypoglycemia for glucose and in nine patients with hypoglycemia /13 patients without hypoglycemia for C‐peptide.

*Note:* Malabsorption/maldigestion comorbidities included: celiac or gluten intolerance; inflammatory bowel disease; lactose intolerance; small intestinal bacterial overgrowth.

Abbreviations: AUC, area under the curve; BMI, body mass index; CD/GI, celiac or gluten intolerance; CFTR, cystic fibrosis transmembrane regulator; HbA1c, hemoglobin A1c; HOMA‐IR, homeostatic model assessment of insulin resistance (using C‐peptide for calculations); IBD, inflammatory bowel disease; IEQ/kg, islet equivalents per kilogram body weight; LI, lactose intolerance; MMTT, mixed meal tolerance test; PRSS1, cationic trypsinogen gene; SIBO, small intestinal bacterial overgrowth; SOD, sphincter of oddi dysfunction; SPINK1, Serine Peptidase Inhibitor, Kazal Type 1; TPIAT, total pancreatectomy with islet autotransplant.

*p‐value compares hypoglycemia to no reported hypoglycemia **p‐value compares level 2 hypoglycemia to no reported hypoglycemia.

Malabsorption or maldigestion comorbidities (excluding pancreas exocrine insufficiency) were over 3‐fold more frequent in the hypoglycemia group (33% vs. 9.9%, *p* < 0.001). Small intestinal bacterial overgrowth (SIBO) represented the most frequently identified condition in both groups, occurring in 43% of cases and 40% of controls with malabsorption disease. Chronic opioid use pre‐surgery did not differ significantly but was more common in those who developed hypoglycemia (67% of cases vs. 52% of controls, *p* = 0.1).

Preoperative islet function and glycemic control laboratory assessments were largely similar between groups, except for a trend towards higher 2 h stimulated C‐peptide in patients with hypoglycemia compared to those without (4.6 vs. 3.6, *p* = 0.059). Glycemic measures including HbA1c and MMTT glucoses, and insulin sensitivity by HOMA‐IR did not differ significantly between cohorts. Transplant‐related characteristics including islet mass transplanted, transplant site, and pylorus resecting versus sparing approach were also comparable between groups. The mean islet yield per kilogram body weight did not differ significantly between groups. Of note, those with hypoglycemia were less likely to have microbial contamination of the islet product than the controls (16% positive culture vs. 32% positive culture, *p* = 0.042).

### 3.4 Pre‐TPIAT and Surgical Characteristics Associated with Level 2 Hypoglycemia

Because some glucose excursions below 70 mg/dL can be observed in a healthy population, we also performed a sensitivity analysis restricting our cases to those with level 2 (< 54 mg/dL) hypoglycemia (*N* = 32) for a stricter definition of hypoglycemia, compared to nonhypoglycemia controls Table 1. This analysis showed the same overall trends the original analysis comparing any hypoglycemia (level 1 or 2) to no hypoglycemia, but with generally increased statistical significance. Those with level 2 hypoglycemia continue to be older (*p* = 0.025), with fewer pediatric TPIAT patients (*p* = 0.007), with a higher BMI (*p* < 0.001), and different etiologies of pancreatitis than controls (*p* = 0.019). Of note, when looking at genetic disease, PRSSS1 and SPINK1 genetic disease was rare among those who developed level 2 hypoglycemia compared to those with no reported hypoglycemia (*p* = 0.025 and 0.046 respectively). Malabsorptive/maldigestion comorbidities remained more common in cases (*p* = 0.002), and gastroparesis emerged as a new association with level 2 hypoglycemia (reported in 56% vs. 35% of controls). In this cohort, opioid use was more common than in controls (78% vs. 52%, *p* = 0.009), and the higher 2 h C‐peptide levels were nearly significant (*p* = 0.052). Surgical associations were similar to the overall cohort.

### 3.5 Comparison of Post‐TPIAT Metabolic Testing in Hypoglycemia and nonhypoglycemia Cohorts

MMTT labs were obtained at approximately 2 years posttransplant (mean 2.25 ± 0.76 years) for patients without reported hypoglycemia; and at the time of hypoglycemia onset (mean 2.96 ± 2.48 years) for patients with hypoglycemia Table 2. MMTT glucoses and C‐peptide, HbA1c, and HOMA‐IR were similar between groups (all *p* > 0.05). The mean BMI was 22.8 ± 3.5 kg/m^2^ versus 21.9 ± 4.9 kg/m^2^ in controls (*p* = 0.2). These results were similar when restricted to the level 2 hypoglycemia cohort, although the evidence of a higher BMI at post‐operative testing in the level 2 hypoglycemia cohort was stronger, but still not statistically significant (23.4 ± 3.6 kg/m^2^ vs. 21.9 ± 4.9 kg/m^2^, *p* = 0.059).

**TABLE 2:** Metabolic testing results after TPIAT in insulin independent patients with hypoglycemia (<70 mg/dl) or level 2 hypoglycemia (<54 mg/dl) versus no reported hypoglycemia post‐TPIAT. Data are displayed as mean (SD). Tests were abstracted from time of reported onset of hypoglycemia in patients with hypoglycemia (2.74 ± 2.29 years post‐TPIAT for HbA1c and 2.96 ± 2.48 years post‐TPIAT for MMTT and fasting glucose and C‐peptide) and at approximately 2 years annual follow up in patients with no reported hypoglycemia (2.21 ± 0.77 years post‐TPIAT for HbA1c and 2.25 ± 0.76 years post‐TPIAT for MMTT and fasting glucose and C‐peptide).

Metabolic tests	No reported hypoglycemia *N* = 111	Hypoglycemia (Level 1 or Level 2) *N* = 45	*p*‐value^*^	Level 2 hypoglycemia *N* = 32	*p*‐value^**^
**HbA1c level (%)**	5.78 (0.67)	5.66 (0.43)	0.2	5.66 (0.42)	0.2
**MMTT results**					
Fasting glucose (mg/dL)	96 (11)	99 (19)	0.3	97 (15)	0.8
1 h glucose (mg/dL)	124 (32)	120 (32)	0.5	127 (32)	0.7
2 h glucose (mg/dL)	100 (23)	96 (22)	0.3	96 (24)	0.4
2 h AUC glucose (mg/dL^*^h)	222 (41)	218 (40)	0.6	223 (40)	>0.9
Any glucose < 70 mg/dL during testing	5 (5.8%)	6 (14%)	0.2	3 (9.7%)	0.4
Fasting C‐peptide (ng/dL)	1.42 (0.70)	1.57 (1.17)	0.4	1.56 (1.27)	0.6
1 h C‐peptide (ng/dL)	3.40 (1.55)	3.94 (2.64)	0.2	4.00 (2.98)	0.3
2 h C‐peptide (ng/dL)	3.11 (1.26)	3.12 (1.31)	>0.9	3.20 (1.37)	0.8
2 h AUC C‐peptide (ng/dL^*^h)	5.65 (2.21)	6.28 (3.02)	0.2	6.38 (3.38)	0.3
**Homa IR**	1.549 (0.027)	1.556 (0.043)	0.3	1.555 (0.047)	0.5

*Note:* Missing laboratory data: HbA1c missing in one participant with hypoglycemia and 14 patients without hypoglycemia; Glucose is missing fasting for fasting for one participant with hypoglycemia and 15 patients without hypoglycemia; 1 h for one participant with hypoglycemia and 25 patients without hypoglycemia; and 2 h for two patients with hypoglycemia and 24 patients without hypoglycemia; C‐peptide is missing fasting for one participant with hypoglycemia and 18 patients without hypoglycemia; 1 h for one participant with hypoglycemia and 24 patients without hypoglycemia; and 2 h for one participant with hypoglycemia and 23 patients without hypoglycemia. AUC calculations could not be completed due to missing data in two patients with hypoglycemia /25 patients without hypoglycemia for glucose and in one participant with hypoglycemia /24 patients without hypoglycemia for C‐peptide.

Abbreviations: AUC, area under the curve; HbA1c, hemoglobin A1c; HOMA‐IR, homeostatic model assessment of insulin resistance (using C‐peptide for calculations); MMTT, mixed meal tolerance test.

*p‐value compares hypoglycemia to no reported hypoglycemia **p‐value compares level 2 hypoglycemia to no reported hypoglycemia.

### 3.6 Hypoglycemia Treatment Approaches and the Response

The majority of patients (35/45, 77.8%) were managed with dietary and lifestyle modifications. CGM was employed in 26 patients (57.8%) either for ongoing glucose monitoring in general, or restarted in response to recurrent hypoglycemic events; an additional eight patients (17.8%) used CGM for short‐term diagnostic evaluation only.

Pharmacologic interventions were utilized in a subset of 15 patients, with some treated with more than one approach. Acarbose was prescribed in 10 cases (22.2%), with four patients reporting gastrointestinal side effects that led to discontinuation. Diazoxide was used in six patients (13.3%), with intolerance reported in one case, resulting in treatment discontinuation. minidose glucagon therapy was initiated in three patients (6.7%); in two patients due to intolerance of other medications, and one used it for exercise‐induced hypoglycemia. One patient was trialed on octreotide during an inpatient hospitalization, which provided no benefit and was not continued in the outpatient setting.

Following treatment, patients perceived and reported a reduced frequency of hypoglycemia 18 cases (40%). Two patients experienced a recurrent severe hypoglycemic event after initiation of therapy. Posttreatment hemoglobin A1c was 5.7 ± 0.4%. Among patients with sufficient follow‐up CGM data for retrospective analyses, nine cases had paired recordings before and after treatment, allowing assessment of the percentage of time spent with glucose levels < 70 mg/dL and < 54 mg/dL. Of those, five cases demonstrated improvement following treatment.

## 4 Discussion

For patients with chronic pancreatitis undergoing TPIAT, about 20%–30% of individuals achieve insulin independence.[2, 3] Spontaneous hypoglycemia has been reported even among insulin independent patients but the frequency, risk factors for, and treatment of hypoglycemia in this population are sparsely reported.[3, 4, 7, 8, 9] In our large cohort of insulin‐independent patients, 28.8% had documented spontaneous hypoglycemic events < 70 mg/dL, and 21% of all insulin independent patients experienced level 2 hypoglycemia (blood glucose < 54 mg/dL). A smaller number reported severe hypoglycemia. Common management strategies included CGM and dietary intervention, with acarbose or diazoxide being the most frequent prescription medications used.

Although hypoglycemia has been previously reported in insulin independent TPIAT, ours is the largest series to examine this phenomenon in insulin independent TPIAT recipients, with over 150 insulin independent patients with long‐term follow up included in our study. Lin et al. previously described this complication in 50% of 12 insulin‐independent individuals within a cohort of 40 TPIAT recipients, but this early study was limited by small sample size with few insulin independent patients.[4] Hypoglycemia was also frequent complaint in our population, with one of every five individuals reporting level 2 hypoglycemia, highlighting the long‐term vulnerability of this population to disordered glucose regulation despite preserved endogenous insulin secretion. These hypoglycemic episodes were clinically significant, with 7% of all insulin‐independent patients in our cohort reporting at least one hypoglycemic episode severe enough to require external assistance, including glucagon administration or medical intervention.

The most common complaints were post‐prandial and exercise induced hypoglycemia, which is similar to previous reports.[4, 6] It is hypothesized that postprandial hypoglycemia may result from postsurgical changes in the gastrointestinal alimentary canal causing an exaggerated incretin effect.[12] Elevated GLP‐1 levels have been documented by our group after TPIAT.[13] Prior work also suggests an impaired glucagon response to falling glucose after a meal in TPIAT which may exacerbate post‐meal hypoglycemia.[14] TPIAT recipients at our center generally have a Roux‐en‐Y reanastomosis of the gastrointestinal tract following pancreaticoduodenectomy and may have reactive hypoglycemia that is similar to that described as a complication of gastric bypass surgery.[12, 15, 16, 17, 18] Exercise‐associated hypoglycemia may be due to an inadequate compensatory response to the increased metabolic demand of activity, with past exercise studies in TPIAT recipients suggesting impaired endogenous hepatic glucose production during moderate exercise.[19, 20] Unlike the earlier small report in the Lin et al. cohort of 12 insulin independent patients, we only rarely observed fasting hypoglycemia;[4] nocturnal hypoglycemia was similarly rare.

Although hypoglycemia after TPIAT can be clinically significant, TP with IAT remains the preferred treatment over TP alone for chronic pancreatitis. Czarnecka et al. reported that while modern insulin therapy and CGM improved glycemic control and safety after TP alone, TPIAT provided superior glycemic control, greater insulin independence, and lower variability, highlighting its metabolic advantage of TPIAT over TP alone. [7] However, our work underscores the importance of monitoring for hypoglycemia even following discontinuation of insulin and understanding this phenomenon to mitigate its clinical impact.

To determine if we could identify risk factors for hypoglycemia in our cohort, we compared demographic, clinical, and surgical characteristics at time of surgery in insulin independent individuals who developed hypoglycemia versus those who did not. We also evaluated glycemic and islet function testing before and after TPIAT in these groups. Patients in the hypoglycemia group were older and more often adults at TPIAT, whereas children were less frequently affected. These results suggest that older or adult recipients may be more susceptible to developing dysregulated glucose control following islet autotransplantation. However, severe hypoglycemia has also been reported in younger patients, as described by Redel et al., who observed this complication in two pediatric cases, so pediatric age should not reduce vigilance in monitoring for this complication.[13] Interestingly, we also observed that cases with hypoglycemia had different etiologies (more idiopathic/less genetic disease), less often had *SPINK1* mutations, and in the case of level 2 hypoglycemia only, less often had PRSS1 mutations. Since these are pancreas specific mutations that should have no impact on post‐TPIAT islet function, we suspect this difference in disease etiology results from fewer children (who have more hereditary disease) in the hypoglycemia cohort.

Notably, the hypoglycemia group had a higher BMI compared with controls, so these patients were not underweight or malnourished in general. It is possible that BMI at the time of surgery or during early postoperative recovery may serve as a surrogate marker of dietary intake patterns. Individuals with higher BMI might consume diets richer in carbohydrates, which could predispose them to greater glycemic excursions and an increased risk of postprandial hypoglycemia. In addition, those who reported other maldigestive/malabsorptive processes were more likely to have hypoglycemia, potentially due to nutrient deficiencies or disordered nutrient absorption, or alternatively this group may have altered their diets in a way that increased risk for hypoglycemia. Within the level 2 hypoglycemia group only, chronic opioid use pre‐TPIAT was more common in the hypoglycemia group, raising the possibility of pituitary–adrenal suppression secondary to chronic opioid use, which has been proposed as a possible mechanism [21]. However, diagnoses of adrenal insufficiency were uncommon and often questionable.

Surgical characteristics potentially influencing glycemic outcomes did not differ significantly between groups. Previous literature suggests that alterations in nutrient flow and vagal innervation following Roux‐en‐Y reconstruction may contribute to post‐TPIAT hypoglycemia, similar to mechanisms observed after bariatric surgery.[5, 17] In our cohort, nearly all patients underwent Roux‐en‐Y reconstruction; pylorus resection was not significantly associated with hypoglycemia but did trend towards more common in the hypoglycemia group. Although prior studies suggest that islet implantation sites may affect glucagon responsiveness, extrahepatic islets in our cohort did not confer protection against hypoglycemia. Likely this is because there are other mechanisms unrelated to glucagon secretion, including impact of enteroendocrine hormone responses after the duodenectomy and Roux‐en‐Y reconstruction. [2, 17, 18, 19] Interestingly, positive islet culture results were more frequent among controls, a finding not readily explained by current evidence.

We did not see differences in metabolic laboratory measures preoperatively or 2–3 years following TPIAT, including HbA1c, HOMA‐IR, and mixed‐meal test results, between the hypoglycemia and control groups. However, asymptomatic hypoglycemia was observed in 25–30% of chronic pancreatitis patients even before surgery and was present in a small subset of controls who did not report hypoglycemia at home after TPIAT. This suggests that patients with chronic pancreatitis are more prone to hypoglycemia for other reasons related to CP such as exocrine pancreas insufficiency. Postoperatively, this phenomenon may become more apparent as patients monitor

There remains limited evidence to guide optimal management strategies for hypoglycemia in this population. Among the various management strategies, dietary modification and the use of CGM were the most commonly employed approaches in our cohort, consistent with previous reports identifying these as first line and often effective interventions. [4, 22] In addition, several patients in our cohort were treated with pharmacologic agents such as acarbose, diazoxide, or minidose glucagon. Although these therapies have been described previously, reports are largely limited to isolated case studies.[8, 10] Acarbose is only sparsely described in TPIAT but is used commonly in gastric bypass and dumping syndrome.[11, 18, 23] Notably, diazoxide has not been reported for management of hypoglycemia following TPIAT; however, its use has been documented in post–bariatric surgery hypoglycemia, particularly after Roux‐en‐Y gastric bypass, with symptomatic improvement.[15, 24]

Although CGM is now routinely implemented in clinical practice, its availability was inconsistent among earlier cases in this retrospective cohort (which dates back to 2010), and reliable data retrieval was not always feasible. Furthermore, limited insurance coverage remains a significant barrier, as many third‐party payers do not cover CGM use for insulin‐independent individuals. These factors—as well as lack of accessibility of complete CGM data in the medical records in some cases– limited our ability to comprehensively assess treatment responses, a challenge that has also been noted in previous studies.[4]

A limitation of our study is the potential for unrecognized asymptomatic hypoglycemic events among insulin‐independent patients who discontinued routine ambulatory glucose monitoring, which may have led to underreporting of hypoglycemia. Additionally, the relatively small sample size, the retrospective nature of the data analyses and medical records data abstraction, and inconsistent access to CGM data further constrained our ability to fully characterize this subgroup or assess treatment responses. Moreover, CGM‐detected hypoglycemia was often brief, as users were alerted and treated promptly, potentially underestimating event duration and severity. Because even healthy individuals can have brief episodes of glucose under 70 mg/dL, some of our patient‐reported hypoglycemia may be ‘normal’.[25] However, most had level 2 hypoglycemia, and association of clinical variables with hypoglycemia was strengthened when we restricted to the cohort who had level 2 hypoglycemia, who represented over 20% of our insulin independent population. Because this study relied upon clinical reports from electronic medical records, we did not have sufficient information in most cases to determine whether the measurements were from CGM alone or confirmed with glucometer. Therefore, it is possible the CGM may have been falsely indicated an episode of hypoglycemia due to known limitations of CGM including compression lows or false reading after sensor start, thereby contributing to overreporting of hypoglycemic events.

Future research is warranted to better identify individuals at risk for hypoglycemia following TPIAT and to develop proactive preventive strategies, as well as evidence‐based management guidelines. Studies involving larger cohorts and more prospective data, incorporating CGM, behavioral diaries to capture lifestyle risk factors such as diet and activity, patient counseling, and standardized assessment of hypoglycemia awareness would be particularly valuable.

## 5 Conclusion

In conclusion, our study demonstrates that spontaneous hypoglycemia is a relatively frequent and clinically meaningful complication among insulin‐independent TPIAT recipients. Even when restricted to documented level 2 hypoglycemia, over 20% of insulin independent patients report hypoglycemia as a concern. Although the exact mechanisms remain incompletely understood, factors such as higher BMI, older age, and malabsorptive disorders may contribute to increased vulnerability, whereas transplant‐related factors appear to play less direct roles. Despite these episodes often arising years after transplantation, consistent monitoring and early intervention, particularly through dietary strategies and CGM use, and help mitigate their impact. Larger, prospective studies integrating metabolic, behavioral, and genetic assessments are needed to refine risk stratification, enhance long‐term surveillance, and guide the development of targeted preventive and therapeutic strategies for this complex posttransplant complication.

## Author Contributions

Concept/design (Melena D. Bellin, Azza Hassaan); Data collection/abstraction (Azza Hassaan, Niveditha Jagadesh, Melena D. Bellin, Guru Trikudanathan, Martin Freeman, Elissa Downs, Srinath Chinnakotla, David Martin, Karthik Ramanathan, Gregory J. Beilman); Data analysis (Anne Eaton); First draft (Azza Hassaan, Anne Eaton, Melena D. Bellin), critical revisions (Niveditha Jagadesh, Guru Trikudanathan, Martin Freeman, Elissa Downs, Srinath Chinnakotla, David Martin, Karthik Ramanathan, Gregory J. Beilman); Final draft (Melena D. Bellin); Funding (Melena D. Bellin,Anne Eaton).

## Conflicts of Interest

The authors do not have a conflict of interest.

## Funding

This work was supported in part by the NIDDK (R01DK126728; PI Bellin).

## Disclosures

MDB receives product research support from Dexcom, serves on a data monitoring board for Vertex, and has consulting or advisory board relationships with Gondola Bio, NovoNordisk, Sana, and Sernova.

## Data Availability

The data that support the findings of this study are available on request from the corresponding author. The data are not publicly available due to privacy or ethical restrictions.
